# Nomogram Predicting the Survival of Young-Onset Patients with Colorectal Cancer Liver Metastases

**DOI:** 10.3390/diagnostics12061395

**Published:** 2022-06-04

**Authors:** Xiaofei Cheng, Yanqing Li, Dong Chen, Xiangming Xu, Fanlong Liu, Feng Zhao

**Affiliations:** 1 Department of Colorectal Surgery, The First Affiliated Hospital, Zhejiang University School of Medicine, Hangzhou 310003, China; xfcheng@zju.edu.cn (X.C.); cdking@zju.edu.cn (D.C.); zjxxm1974@163.com (X.X.); 2 Department of Pathology, The Second Affiliated Hospital, Zhejiang University School of Medicine, Hangzhou 310009, China; 2512074@zju.edu.cn; 3 Department of Radiation Oncology, The First Affiliated Hospital, Zhejiang University School of Medicine, Hangzhou 310003, China

**Keywords:** colorectal cancer liver metastases (CRLM), young-onset, prognosis factors, nomogram

## Abstract

**Background:** Although the global prevalence of colorectal cancer (CRC) is decreasing, there has been an increase in incidence among young-onset individuals, in whom the disease is associated with specific pathological characteristics, liver metastases, and a poor prognosis. **Methods:** From 2010 to 2016, 1874 young-onset patients with colorectal cancer liver metastases (CRLM) from the Surveillance, Epidemiology, and End Results (SEER) database were randomly allocated to training and validation cohorts. Multivariate Cox analysis was used to identify independent prognostic variables, and a nomogram was created to predict cancer-specific survival (CSS) and overall survival (OS). Receiver operating characteristic (ROC) curve, C-index, area under the curve (AUC), and calibration curve analyses were used to determine nomogram accuracy and reliability. **Results:** Factors independently associated with young-onset CRLM CSS included primary tumor location, the degree of differentiation, histology, M stage, N stage, preoperative carcinoembryonic antigen level, and surgery (all *p* < 0.05). The C-indices of the CSS nomogram for the training and validation sets (compared to TNM stage) were 0.709 and 0.635, and 0.735 and 0.663, respectively. The AUC values for 1-, 3-, and 5-year OS were 0.707, 0.708, and 0.755 in the training cohort and 0.765, 0.735, and 0.737 in the validation cohort, respectively; therefore, the nomogram had high sensitivity, and was superior to TNM staging. The calibration curves for the training and validation sets were relatively consistent. In addition, a similar result was observed with OS. **Conclusions:** We developed a unique nomogram incorporating clinical and pathological characteristics to predict the survival of young-onset patients with CRLM. This may serve as an early warning system allowing doctors to devise more effective treatment regimens.

## 1. Introduction

Colorectal cancer (CRC) is the third most common malignancy and leading cause of cancer death in both men and women worldwide [1]. Since the mid-2000s, the incidence of CRC in both sexes in the United States has fallen by 2–3% each year because of the widespread use of screening tests that allow detection and excision of pre-malignant lesions [1,2]. However, the incidence of young-onset CRC, defined as CRC developing before the age of 50 years, has risen in recent years [3]. Adult CRC survival improved from 1973 to 2005, whereas child and adolescent survival did not [4,5]. Compared to the elderly, young patients are more prone to distant metastases and microsatellite instability, both of which are linked to poor outcomes [6]. Young-onset CRC causes both financial loss and loss of life. Appropriate therapies for young-onset CRC patients are lacking.

In individuals with CRC, the liver is the most common site of metastatic disease. In 20–25% of patients in whom colorectal cancer is identified for the first time, liver metastases are also found [7]. Younger CRC patients have more liver metastases than older CRC patients, possibly due to delays in diagnosis [8,9]. No consensus has emerged on whether the colorectal metastases of young people are identical to the colorectal cancer liver metastases (CRLM) of older patients, or a unique molecular/immunological entity. Due to variation in genetic, cultural, nutritional, and regional factors, it is difficult to predict the long-term survival of young-onset patients with CRLM. Currently, both the American Joint Committee on Cancer (AJCC), TNM stage, and clinical experience inform predictions of the CRLM prognosis and survival of young-onset patients. However, the TNM stage considers only a few criteria, and many clinical features that affect prognosis are overlooked [10]. Reliable prognostic predictions are crucial when selecting therapy, and to ensure good communication between clinicians and young-onset CRLM patients.

Often, nomograms that consider many independent predictors of survival are more accurate, and more intuitive when applied clinically, than other survival prediction methods. We used the Surveillance, Epidemiology, and End Results (SEER) database to acquire information on CRLM in young-onset patients. All cases were separated into training and validation sets. Using common clinicopathological criteria, we developed an efficient and precise nomogram predicting CRLM prognosis in young-onset patients, and a histogram that assessed predictive power.

## 2. Materials and Methods

### 2.1. Patients 

**Figure 1:**
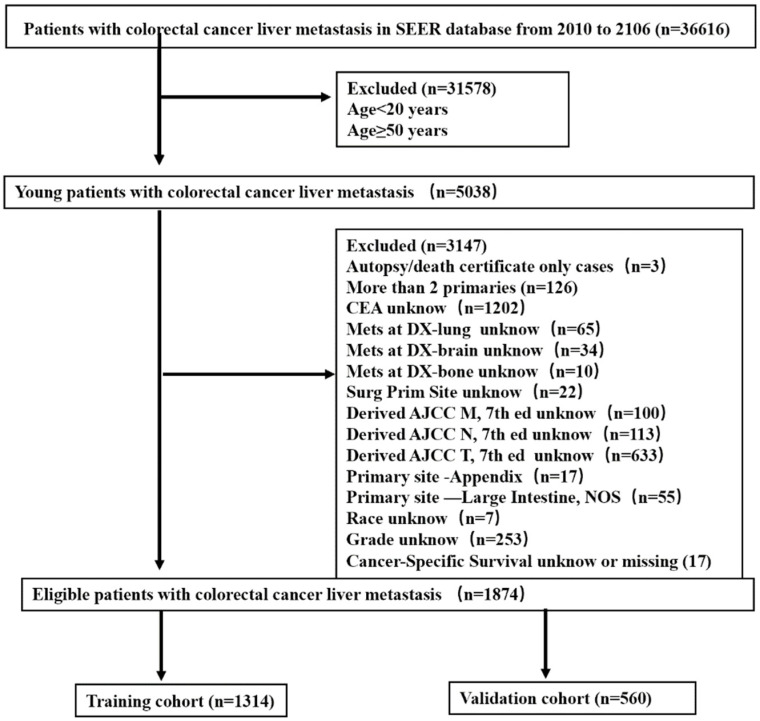
Strategies for selecting patients to be included in the study.

We identified 1874 young-onset CRLM patients in the SEER database using the following selection criteria: aged 20–49 years, and CLRM evident at the initial diagnosis from 2010 to 2016. Patients diagnosed on the basis of autopsies or death certificates were excluded, as were those without comprehensive information. Figure 1 shows a flow diagram of the patient selection process. SEER database analyses are exempt from medical ethics approval, so no informed patient consent process was necessary.

### 2.2. Data Collection

Data on age, sex, tumor site, degree of differentiation, histological type, TNM stage, T stage, N stage, M stage, carcinoembryonic antigen (CEA) level, and primary and metastatic surgery status were retrieved. The seventh edition of the AJCC staging system was used to classify all clinicopathological factors. Adenocarcinoma (8010, 8020, 8140–8141, 8144, 8210,8211, 8255, 8260, 8261, 8263, 8310, 8440, 8460, 8550, 8560) and mucinous adenocarcinoma (8470, 8471, 8472, 8480, 8481) were the two histological subtypes of CRC defined by the ICD-O-3 oncology codes. The SEER database was used to determine survival and the ultimate cause of death. For model creation and assessment, training and validation sets were generated.

### 2.3. Statistical Analysis

The chi-squared test and Fisher’s exact test were used to compare categorical variables, which are expressed as numbers with percentages. Multi-factor survival analysis was performed using Cox’s regression, and a nomogram was created. The concordance index (C-index) refers to the proportion of all patient pairs that agreed with the findings. To assess prognostic accuracy, time-dependent receiver operating characteristic (ROC) curves were drawn, and the areas under the curves (AUCs) were calculated at 1, 3, and 5 years. The calibration curve was used to determine if the nomogram-predicted survival probability matched that of 1000-bootstrap resampling. The Kaplan–Meier method and log-rank test were used to examine the survival curves. SPSS (version 25.0; IBM Corp., Armonk, New York, NY, USA) and R software (version 4.12; R Foundation for Statistical Computing, Vienna, Austria) were used to execute all statistical procedures. A *p*-value < 0.05 was regarded as statistically significant.

## 3. Results

### 3.1. Basic Patient Characteristics

A total of 36,616 patients with CRLM were found in the SEER database from 2010 to 2016, including 5038 aged 20–49 years (13.8% of all patients). After rigorous screening, 1874 young-onset patients with liver CRLM were included. The patients were randomly divided into 1314 cases in a training set and 560 cases in a validation set using the random sampling method and a 7:3 ratio based on R software 4.12 (caret package). The two groups did not differ significantly in demographic or clinical variables. Table 1 lists the patient characteristics. 

In total, 54.5% of the patients were male and 33.5% of the tumors were in the rectum. Of all tumors, 75.7% were well- or moderately differentiated. Of all patients, 80.4% were CEA-positive and 93.1% had adenocarcinomas. Furthermore, in 25.8% of patients, both the primary tumor and liver metastases were removed at the same time, while only the primary tumor was removed in 49.9%. Of all patients, hepatic metastases only occurred in 81.1% and extrahepatic metastases in 18.9%.

### 3.2. Independent Features Predictive of Prognosis in Young-Onset Patients with CRLM

Seven characteristics, including the primary tumor site, degree of differentiation, N stage, M stage, histology, surgery, and CEA level were independent predictors of CSS on multivariate Cox’s regression analysis (Table 2).

Patients with undifferentiated or poorly differentiated carcinomas exhibited poorer outcomes (hazard ratio (HR) = 1.938, 95% confidence interval (CI)= 1.637–2.294, *p* < 0.0001) than those with well- or moderately differentiated carcinomas. The prognosis of patients with primary tumors in the left colon (HR = 0.574, 95% CI = 0.476–0.691, *p* < 0.0001) and rectum (HR = 0.614, 95% CI = 0.504–0.748, *p* < 0.0001) was better than that of patients with tumors in the right colon, but there was no significant difference between patients with tumors in the left colon and rectum. N2 stage patients (HR = 1.405, 95% CI = 1.120–1.763, *p* = 0.003) and M1b stage (HR = 1.624, 95% CI = 1.352–1.952, *p* < 0.0001) exhibited a poorer prognosis than N0 and M1a stage patients. The prognosis of CEA-negative patients was better than that of CEA-positive patients (HR = 0.773, 95% CI =0.633–0.943, *p* = 0.011). Patients who underwent resection of both the primary and metastatic sites (HR = 0.378, 95% CI = 0.291–0.490, *p* < 0.0001) or primary site alone (HR = 0.567, 95% CI = 0.452–0.712, *p* < 0.0001) had a better prognosis than those who did not undergo resection of both sites, but resection of the liver alone did not provide a survival benefit. In addition, a similar result was observed with OS (Table S1).

### 3.3. Construction of the Nomogram

**Figure 2:**
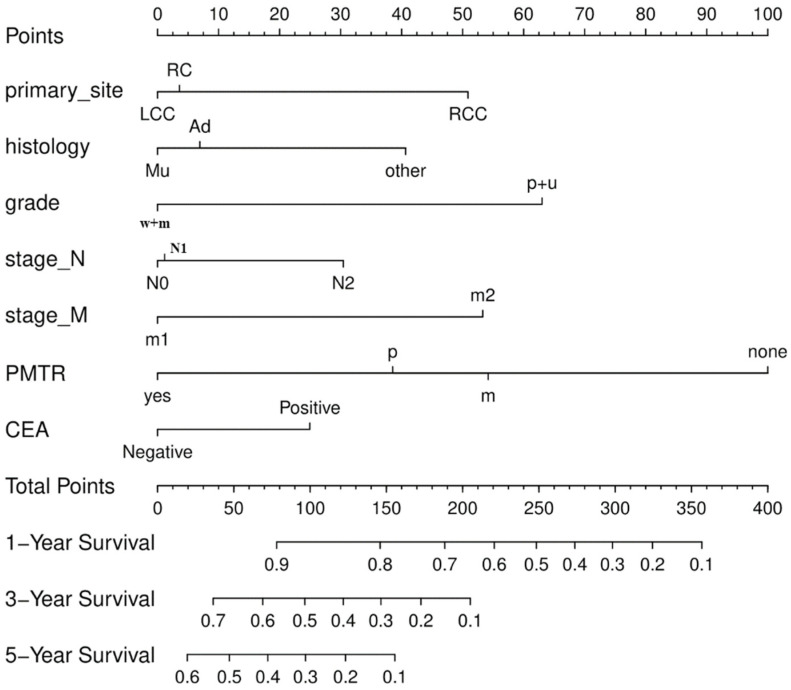
The Nomogram for predicting cancer-specific survival (CSS). Right colon cancer (RCC); Left colon cancer (LCC); Rectal cancer (RC); Well + Moderately (w + m); Poorly + Undifferentiated (p + u); Surgery (PTMR); Neither (none); Primary+ Metastatic (yes); Only primary (p); Only metastatic (m).

We used multivariable Cox’s regression analysis to create a nomogram that considered the seven main factors affecting survival. The top ruler of the nomogram is used to calculate a risk score for each variable; the probability of 1-, 3-, and 5-year CSS is determined by superimposing the risk score for each variable on the bottom ruler (Figure 2).

A prognostic nomogram for OS in young-onset patients with CRLM is presented in Figure S1.

### 3.4. Validation of the Nomogram

For the training set, the CSS nomogram had a C-index of 0.709 (95% CI = 0.689–0.729), indicating high accuracy in terms of CSS prediction. For both the training and validation sets, the C-indices of the CSS nomogram were higher than those of the TNM stage (Table 3). 

**Figure 3:**
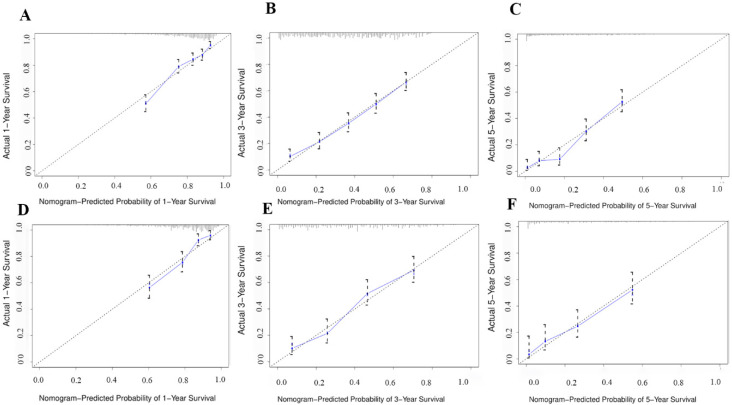
Calibration plots of the nomogram for 1-, 3-, and 5-year CSS prediction in the training set (**A**–**C**) and validation set (**D**–**F**).

The CSS nomogram was well-calibrated, with the mean projected probability for each subgroup being similar to the observed probability, as revealed by the calibration plots (Figure 3A–F).

**Figure 4:**
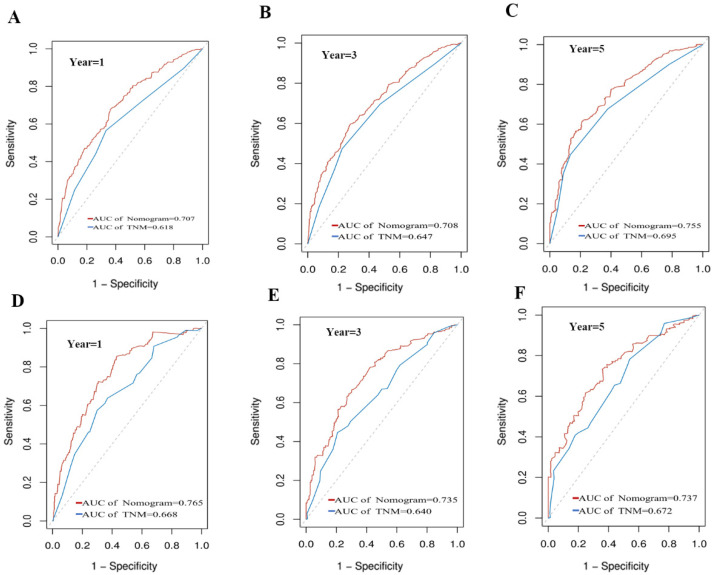
Comparison of the ROC curves of the nomogram and the TNM stage for 1-, 3-, and 5-year CSS prediction in the training set (**A**–**C**) and validation set (**D**–**F**).

We generated ROC curves to assess the survival predictions. The predictive accuracy of the CSS nomogram was better than that of the TNM stage in both the training and validation sets. When the CSS nomogram was compared to the TNM stage in terms of the training set, the 1-, 3-, and 5-year AUC values were 0.707 and 0.618, 0.708 and 0.647, and 0.755 and 0.695, respectively (Figure 4A–C). For the validation set, the respective values were 0.765 and 0.668, 0. 735 and 0.640, and 0.737 and 0.672 (Figure 4D–F). 

**Figure 5:**
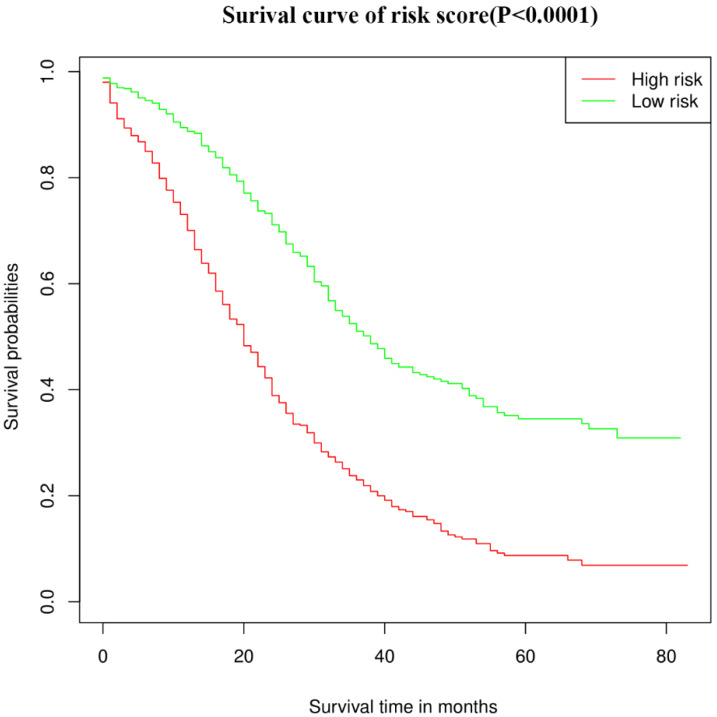
Kaplan–Meier survival curve of the CSS in the high- and low-risk groups.

We calculated the relative risk coefficient for young-onset patients with CRLM based on the multivariate COX regression risk scale model. Patients with relative risk coefficients greater than the median were defined as the high-risk group, while patients with relative risk coefficients less than the median were defined as the low-risk group. The high-risk group had a median survival of 20 months, whereas the low-risk group had a median survival of 38 months. Figure 5 shows the survival curves (*p* < 0.0001). In addition, the time-dependent ROC results showed that the prediction accuracy of the OS nomogram was better than the TNM stage in both training and validation set (Figures S2 and S3).

## 4. Discussion

Distant organ metastasis (mainly to the liver) is a clinical hallmark of young-onset CRC, accounting for most of the deaths [11]. Multiple liver metastases, nodules, the degree of differentiation, extrahepatic metastasis, tumor size, CEA level, positive surgical margins, and venous infiltration are all significant predictors of CRLM patient survival [12,13,14]. However, clinicopathological characteristics differ significantly between younger and older CRC patients. Young-onset CRC tends to be more advanced at diagnosis, and exhibits poorer cell differentiation and a higher likelihood of signet ring cell histology; moreover, the primary tumor is more likely to be on the left side of the colon [15]. It is unclear whether models predicting CRLM prognosis in the general population are appropriate for young-onset patients. Therefore, we identified risk factors for younger patients and developed a model predicting survival based on specific pathological tumor characteristics. We created a nomogram that combined clinicopathological factors with the TNM stage to predict survival in young-onset CRLM patients. The primary tumor site and grade, N and M stages, pretreatment CEA level, histology, and resection of primary or metastatic sites were associated with prognosis. The nomogram was validated and calibrated by identifying the most important parameters, and has the potential for wide application. In terms of both the ROC analysis and C-index, the nomogram outperformed the TNM staging method in terms of predictive accuracy and prognostic utility. The survival curve indicated that the low-risk group had a much better prognosis.

The primary tumor site significantly affected the clinical outcome [16]. Young-onset CRLM patients with rectal and left colon tumors had better outcomes than those with right colon tumors, but there was no significant difference between patients with rectal and left colon tumors. According to a previous study based on the SEER database, CRLM patients with colon primaries had worse survival than those with rectal primaries [14]. Different from our findings, this suggests that young-onset CLRM patients may have unique prognostic factors. Right-sided CRC has a worse prognosis than left-sided CRC, as evidenced by the higher prevalence of mucinous, undifferentiated, and signet-ring cell tumors, and more advanced disease at diagnosis [17,18]. Patients with right-sided colon tumors and CRLM had a lower 5-year OS rate after surgery than those with left-sided colon tumors [19]. More studies are needed to determine whether the primary tumor site affects survival differently between younger patients with CRLM and older patients.

CEA is a cell surface glycoprotein expressed by normal mucosal cells, but is overexpressed in cancers. The CEA level was useful for predicting CRLM patient outcomes [10,20,21]. We also found that CEA-positive young-onset CRLM patients had a worse prognosis than CEA-negative patients. Furthermore, the lower the degree of differentiation of tumor cells, the poorer the survival. CEA-positive patients with a low degree of tumor differentiation must be closely monitored after discharge. The OS and cancer-specific survival nomograms, and tumor survival risk scores for the T1 stage, are higher than those for T2–T3 stage patients with CRLM, indicating that T1 tumors are associated with poorer survival [10,22]. However, we found that T stage had no significant effect on the survival of young-onset patients with CRLM. This difference in the effect of T stage by age requires further examination.

CRLM surgery remains contentious [23]. The most effective curative treatment for patients with CRLM is radical resection of the initial tumor combined with removal of liver metastases [24,25,26]. The median survival time of our young-onset CRLM patients who underwent resection of both the primary and metastatic sites was 38 months, which was significantly longer than that of patients who did not undergo surgery (18 months). For patients with unresectable liver metastases, the survival benefit afforded by resection of the primary lesion alone remains controversial. Primary tumor excision enhances quality of life and minimizes the adverse effects of systemic chemotherapy, as well as the risk of primary tumor complications (bleeding, blockage, and perforation) [27,28]. However, primary tumor excision delays systemic chemotherapy, particularly if complications emerge [29]. A multicenter retrospective cohort study showed that primary tumor excision significantly increased OS in patients with stage IV CRC and unresectable metastases [30,31]. We found that young-onset CRLM patients who underwent only primary resection had a median survival time of 26 months, which was much longer than that of patients who did not undergo surgery. This indicates that we should take a more aggressive approach to the management of the primary site in young-onset patients with CRLM.

In this study, we examined common clinicopathological features, individually and in combination, to develop a simple, quick, and accurate predictive model. However, the study had several limitations. First, the SEER database lacks information on the frequency and extent of liver metastases, mutations, and CA199 status. Second, apart from surgeries, the database lacked treatment information. Finally, our nomogram remains to be externally validated.

In conclusion, our nomogram correctly predicted the survival of young-onset CRLM patients, showed good discrimination and calibration, and will allow physicians to individualize prognoses and explore therapeutic solutions for young-onset CRLM patients.

## Figures and Tables

**Table 1:** Patient demographics and pathological characteristics.

Variable	All Patients (*n* = 1874)		Training Cohort (*n* = 1314)		Validation Cohort (*n* = 560)		*p* Value
	No.	%	No.	%	No.	%	
**Race**							0.678
Black	290	15.5	204	15.5	86	15.4	
White	1372	73.2	956	72.8	416	74.3	
Other	212	11.3	154	11.7	58	11.3	
**Sex**							0.269
Female	853	45.5	609	46.3	244	43.6	
Male	1021	54.5	705	53.7	316	56.4	
**Primary site**							0.369
RCC	537	28.7	384	29.2	153	28.7	
LCC	708	37.8	502	38.2	206	37.7	
RC	629	33.5	428	32.6	201	33.6	
**Histology**							0.513
Adenocarcinoma (Ad)	1744	93.1	1218	92.7	526	93.9	
Mucinous adenocarcinoma (Mu)	90	4.8	68	5.2	22	3.9	
Other	40	2.1	28	2.1	12	2.1	
**Grade**							0.975
Well + moderately (w + m)	1418	75.7	994	75.6	424	75.7	
Poorly + undifferentiated (p + u)	456	24.3	320	24.4	136	24.3	
**Stage_T**							0.137
T0-1	182	9.7	131	10.0	51	9.1	
T2	63	3.4	36	2.7	27	4.8	
T3	1020	54.4	721	54.9	299	53.4	
T4	609	32.5	426	32.4	183	32.7	
**Stage_N**							0.690
N0	393	21.0	274	20.9	119	21.3	
N1	767	40.9	546	41.6	221	39.5	
N2	714	38.1	494	37.6	220	39.3	
**Stage_M**							0.170
M1a	1142	60.9	814	61.9	328	58.6	
M1b	732	39.1	500	38.1	232	41.4	
**Surgery (PTMR)**							
Neither (none)	424	22.6	286	21.8	138	24.6	0.201
Primary+ metastatic (yes)	483	25.8	345	26.3	138	24.6	
Only primary (p)	936	49.9	657	50.0	279	49.8	
Only metastatic (m)	31	1.7	26	2.0	5	0.9	
**Extrahepatic metastasis (met)**							0.290
No	1520	81.1	1074	81.7	446	79.6	
Yes	354	18.9	240	18.3	114	20.4	
**CEA**							0.175
Positive	1507	80.4	1046	79.6	461	82.3	
Negative	367	19.6	268	20.4	99	17.7	

**Table 2:** Multivariable analyses of Cancer-Specific Survival in the training cohort.

Variable	Multivariate Analysis		
	HR	95% CI	*p* Value
**Race**			
Black	Reference		
White	0.859	0.703–1.049	0.135
Other	0.985	0.746–1.300	0.914
**Sex**			
Female	Reference		
Male	1.065	0.917–1.238	0.407
**Primary site**			
RCC	Reference		
LCC	0.574	0.476–0.691	<0.0001
RC	0.614	0.504–0.748	<0.0001
**Histology**			
Adenocarcinoma (Ad)	Reference		
Mucinous adenocarcinoma (Mu)	0.938	0.672–1.309	0.704
Other	1.578	1.008–2.471	0.046
**Grade**			
Well + moderately (w + m)	Reference		
Poorly + undifferentiated (p + u)	1.938	1.637–2.294	<0.0001
**Stage_T**			
T0-1	Reference		
T2	0.793	0.452–1.392	0.419
T3	0.781	0.596–1.023	0.073
T4	1.042	0.794–1.368	0.766
**Stage_N**			
N0	Reference		
N1	1.019	0.829–1.254	0.856
N2	1.405	1.120–1.763	0.003
**Stage_M**			
M1a	Reference		
M1b	1.624	1.352–1.952	<0.0001
**Surgery (PTMR)**			
Neither (none)	Reference		
Primary+ metastatic (yes)	0.378	0.291–0.490	<0.0001
Only primary	0.567	0.452–0.712	<0.0001
Only metastatic	0.642	0.363–1.133	0.126
**Extrahepatic metastasis (met)**			
No	Reference		
Yes	1.222	0.982–1.521	0.072
**CEA**			
Positive	Reference		
Negative	0.773	0.633–0.943	0.011

**Table 3:** C-indexes for the nomograms and TNM stage.

Survival	Training Cohort			Validation Cohort		
	C-Index	95% CI		C-Index	95% CI	
Nomogram	0.709	0.689	0.729	0.735	0.708	0.762
TNM stage	0.635	0.613	0.657	0.663	0.629	0.696

## Data Availability

The data that support the findings of this study are openly available in the Surveillance, Epidemiology, and End Results (SEER) database of the National Cancer Institute at https://seer.cancer.gov/ (accessed on 15 November 2021).

## Supplementary Materials

The following supporting information can be downloaded at https://www.mdpi.com/article/10.3390/diagnostics12061395/s1. Table S1: Multivariable analyses of overall survival in the training cohort, Figure S1: The Nomogram for predicting overall survival (OS), Figure S2: Calibration plots of the nomogram for 1-, 3-, and 5-year OS prediction in the training set (A–C) and validation set (D–F), Figure S3: Comparison of the ROC curves of the nomogram and the TNM stage for 1-, 3-, and 5-year OS prediction in the training set (A–C) and validation set (D–F).
