# Supplementary material for: Nomogram Predicting the Survival of Young-Onset Patients with Colorectal Cancer Liver Metastases
Source: Diagnostics (Basel). 2022 Jun 4;12(6):1395. doi: 10.3390/diagnostics12061395 (PMC9221975; doi:10.3390/diagnostics12061395)
Supplement: Supplementary file 1 [file diagnostics-12-01395-s001.zip › diagnostics-1739612-supplementary.pdf]

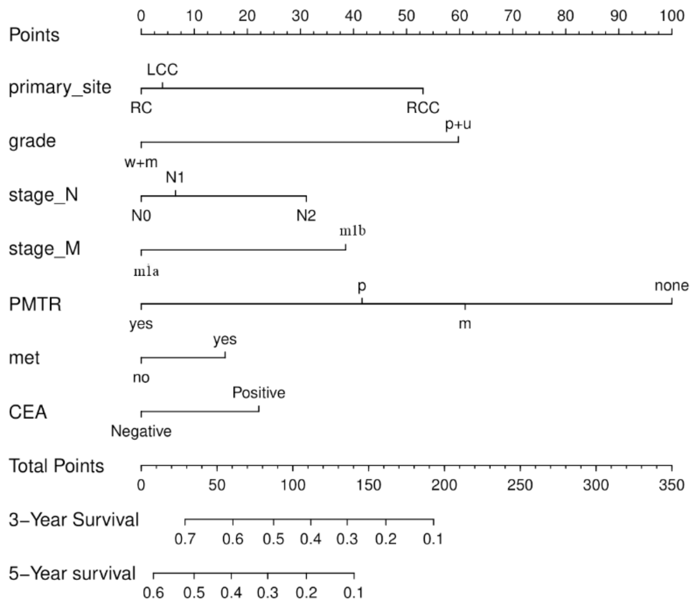


Figure S1. The Nomogram for predicting overall survival (OS).

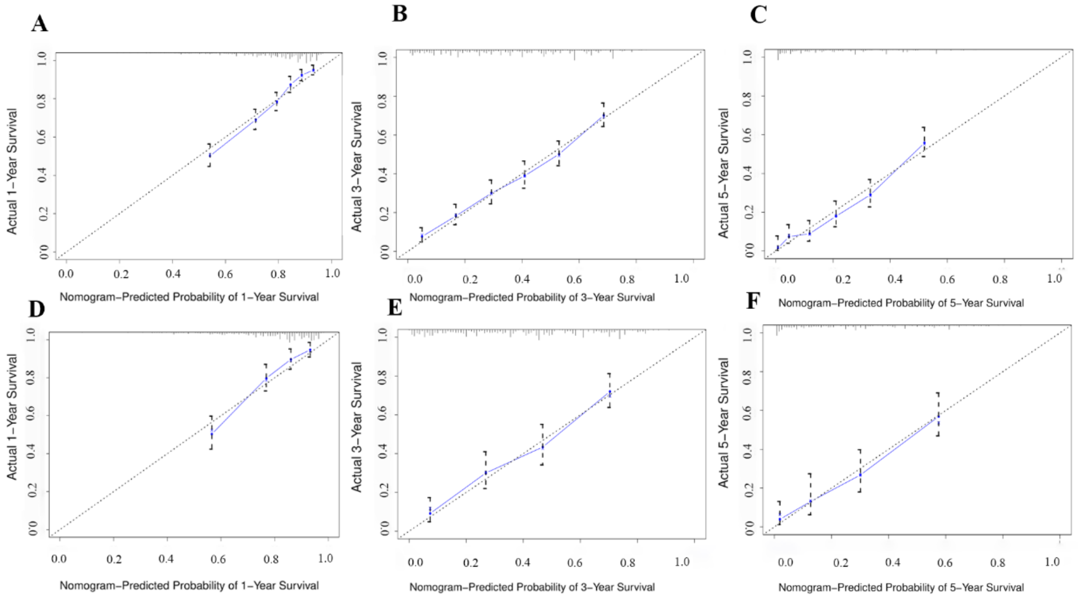


Figure S2: Calibration plots of the nomogram for 1-, 3-, and 5-year OS prediction in the training set (A–C) and validation set (D–F)

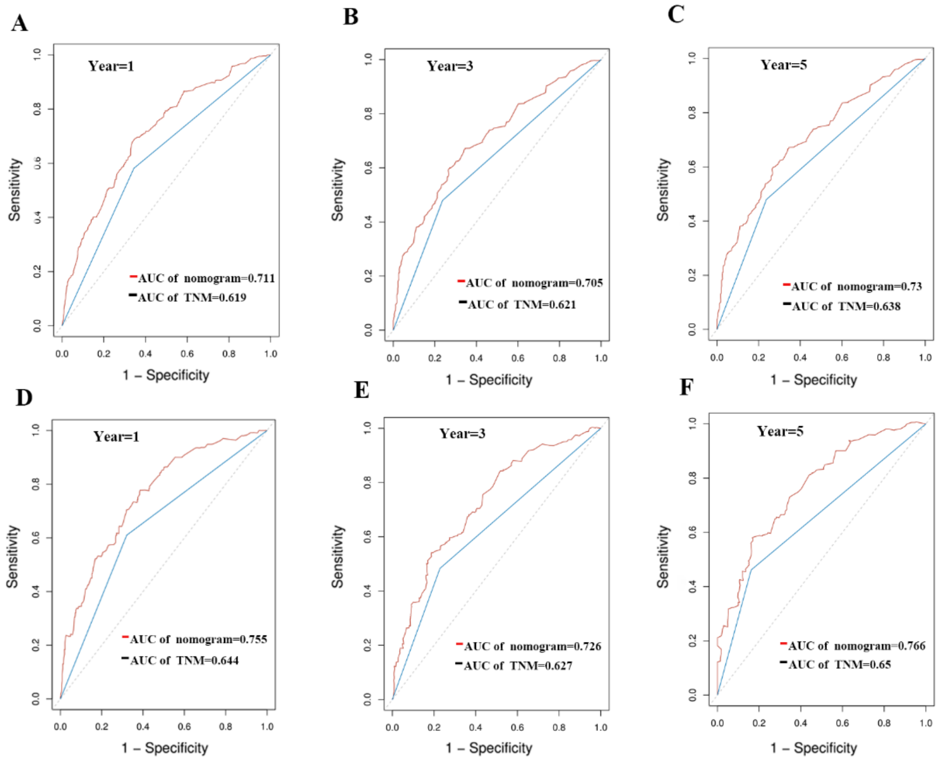


Figure S3: Comparison of the ROC curves of the nomogram and the TNM stage for 1-, 3-, and 5-year OS prediction in the training set (A–C) and validation set (D–F)

**Table S1 Multivariable analyses of overall survival in the training cohort**

| Variable | Multivariate analysis | | |
|---|---|---|---|
| | HR | 95%CI | p value |
| **Race** | | | |
| black | | Reference | |
| White | 0.900 | 0.745-1.087 | 0.274 |
| other | 0.936 | 0.713-1.229 | 0.633 |
| **Sex** | | | |
| female | | Reference | |
| male | 1.029 | 0.891-1.190 | 0.690 |
| **Primary site** | | | |
| RCC | | Reference | |
| LCC | 0.592 | 0.495-0.709 | <0.0001 |
| RC | 0.590 | 0.485-0.717 | <0.0001 |
| **Histology** | | | |
| Adenocarcinoma (Ad) | | Reference | |
| Mucinous adenocarcinoma (Mu) | 1.000 | 0.709-1.403 | 0.987 |
| Other | 1.441 | 0.911-2.278 | 0.118 |
| **Grade** | | | |
| well+moderately (w+m) | | Reference | |

| | | | |
|---|---|---|---|
| poorly+undifferentiated (p+u) | 1.441 | 1.581-2.181 | <0.0001 |
| **Stage_T** | | | |
| T0-1 | | Reference | |
| T2 | 0.751 | 0.467-1.209 | 0.238 |
| T3 | 0.792 | 0.609-1.029 | 0.081 |
| T4 | 1.029 | 0.788-1.343 | 0.835 |
| **Stage_N** | | | |
| N0 | | Reference | |
| N1 | 1.087 | 0.891-1.325 | 0.411 |
| N2 | 1.430 | 1.151-1.777 | 0.001 |
| **Stage_M** | | | |
| M1a | | Reference | |
| M1b | 1.513 | 1.270-1.803 | <0.0001 |
| **Surgery (PTMR)** | | | |
| Neither (none) | | Reference | |
| primary+ metastatic (yes) | 0.347 | 0.269-0.447 | <0.0001 |
| only primary | 0.556 | 0.444-0.696 | <0.0001 |
| only metastatic | 0.650 | 0.377-1.123 | 0.123 |
| **Extrahepatic metastasis(met)** | | | |
| no | | Reference | |
| yes | 1.202 | 0.975-1.481 | 0.085 |
| **CEA** | | | |
| positive | | Reference | |
| negative | 0.786 | 0.648-0.952 | 0.013 |
